# Role of gender in explaining metabolic syndrome risk factors in an Iranian rural population using structural equation modelling

**DOI:** 10.1038/s41598-023-40485-y

**Published:** 2023-09-25

**Authors:** Marjan Nouri-Keshtkar, Mohadeseh Shojaei Shahrokhabadi, Azadeh Ghaheri, Roya Hosseini, Hassan Ketabi, Mojtaba Farjam, Ding-Geng Chen, Mehdi Rezaeian, Reza Homayounfar, Yaser Tahamtani, Mehdi Totonchi

**Affiliations:** 1 Department of Developmental Biology, University of Science and Culture, Tehran, Iran; 2 Department of Stem Cells and Developmental Biology, Cell Science Research Center, Royan Institute for Stem Cell Biology and Technology, ACECR, Tehran, Iran; 3 Biostatistics Research Group, Population Health Sciences Institute, Newcastle University, Newcastle upon Tyne, UK; 4 Department of Basic and Population Based Studies in NCD, Reproductive Epidemiology Research Center, Royan Institute, ACECR, Tehran, Iran; 5 Department of Andrology, Reproductive Biomedicine Research Center, Royan Institute for Reproductive Biomedicine, ACECR, Tehran, Iran; 6 Mobile Telecommunication Company of Iran, Tehran, Iran; 7 Noncommunicable Diseases Research Center, Fasa University of Medical Sciences, Fasa, Iran; 8 College of Health Solutions, Arizona State University, Phoenix, AZ USA; 9 Department of statistics, University of Pretoria, Pretoria, South Africa; 10 National Nutrition and Food Technology Research Institute, Faculty of Nutrition and Food Technology, Shahid Beheshti University of Medical Sciences, Tehran, Iran; 11 Department of Genetics, Reproductive Biomedicine Research Center, Royan Institute for Reproductive Biomedicine, ACECR, Tehran, Iran

**Keywords:** Endocrinology, Health care, Medical research, Risk factors

## Abstract

Many factors can lead to an increase in the prevalence of metabolic syndrome (MetS) in different populations. Using an advanced structural equation model (SEM), this study is aimed to determine the most important risk factors of MetS, as a continuous latent variable, using a large number of males and females. We also aimed to evaluate the interrelations among the associated factors involved in the development of MetS. This study used data derived from the Fasa PERSIAN cohort study, a branch of the PERSIAN cohort study, for participants aged 35 to 70 years with 10,138 males and females. SEM was used to evaluate the direct and indirect effects, as well as gender effects of influencing factors. Results from the SEM showed that in females most changes in MetS are described by waist circumference (WC), followed by hypertension (HP) and triglyceride (TG), while in males most changes in MetS are described by WC, followed by TG then fasting blood glucose (FBG). Results from the SEM confirmed the gender effects of social status on MetS, mediated by sleep and controlled by age, BMI, ethnicity and physical activity. This study also shows that the integration of TG and WC within genders could be useful as a screening criterion for MetS in our study population.

## Introduction

Metabolic syndrome (MetS) is manifested by insulin resistance, central obesity, dyslipidemia, and hypertension and the diagnosis of MetS is based on the co-occurrence of these risk factors^1^ The presence of the MetS can significantly increase the risk of atherosclerotic cardiovascular disease (ASCVD) and mortality due to this disease as well as the risk of type 2 diabetes^2–4^. Moreover, MetS is associated with the risk of certain types of cancers, such as pancreatic, colorectal, and breast cancer^5–8^.

With the worldwide increasing prevalence of sedentary lifestyle and consequently increasing of obesity^9^, MetS has become a growing public health challenge, with an estimated prevalence of 10–40%^10^. In Iran, according to several systematic review and meta-analysis as well as based on different definitions, the overall prevalence of MetS is estimated approximately 33.7% indicating the relatively high prevalence of this disease in the Iranian population^11^.Although genetic predisposition plays an important role in the regulation of body weight and other metabolic parameters, susceptible individuals are also influenced by physical, social, economic and cultural factors as well^12^.

MetS is usually considered as a binary variable when assessed according to commonly used definitions. For example, according to the US National Cholesterol Education Program Adult Treatment Panel III (NCEP ATP III) criteria, the presence of MetS is defined as having three or more abnormal components of MetS; However, discarding information on how close each MetS component was to the threshold might result in large information loss; Therefore, using a method which enables us evaluate MetS as a continuous latent or a construct variable, which considers the combination of the multiple dimensions of MetS seems more appropriate than using a single observed binary variable for MetS. In this regard, we used the advanced Structural Equation Model (SEM) as a theory-driven data analytical approach. SEM is an efficient technique to evaluate the hypothesized simultaneous direct and indirect relationships among a set of variables, including those that cannot be directly measure, as MetS in this study^13^. Thus, the main purpose of the present study was to use SEM to model MetS risk factors considering the five components as continuous variables that represent the concept of the MetS as a latent continuous variable rather than a binary one we performed the analysis for males and female participants separately to determine which components of MetS are regarded as the most important ones in this Iranian rural population using a large number of males and females. We also evaluated the interrelations among the associated factors involved in the development of MetS employing SEM.

## Method

### Origin of data

The data used in this study was driven from the baseline survey of the Fasa PERSIAN cohort study (FPCA). Fasa PERSIAN cohort study was a part of the Prospective Epidemiological Research Study in IRAN (PERSIAN) cohort study and was conducted in 2014 as a large epidemiological study on a random sample from the general population^14,15^. FPCS was conducted according to the Declaration of Helsinki, and the regional and national research ethics committees of Fasa University of Medical Sciences (FUMS) has approved the study protocol (Ethic code IR.FUMS.RES.1394.3). Written informed consent was obtained from all participants before taking part in the study for examination and interview. In addition, the participants were able to withdraw from the study at any time if they wished to do so.

### Study population

The first phase of PERSIAN population-based cohort was conducted from Oct. 2014 to Sep 2016. A rural region known as Sheshdeh, the suburb of Fasa city and its 24 satellite villages with a total population of 41,000 was chosen for the cohort study and 11,097 adult subjects aged 35 to 70 were recruited into the study.

### Metabolic syndrome assessment and Measurements

MetS components, defined by National Cholesterol Education Program Adult Treatment Panel III (NCEP ATP III)^16^ were measured as follows: (1) Waist circumference (WC) was measured in the standing position at the middle between the lower rib and the iliac crest without any pressure to the body surface; (2) Blood pressure (BP) was measured twice after at least 5 min of rest by using right arm in a sitting position. The average of the two measurements was recorded for both systolic and diastolic BP; (3) Fasting blood glucose (FBG); (4) high-density lipoproteins (HDL)- cholesterol (KIMIA Kit, code 890,303, Iran) and triglycerides (TG) were measured by standard method in a central, certified laboratory using Pars Azmoon kits (Pars Azmoon Co., Tehran, Iran). According to joint NCEP ATP III criteria and Iranian-specific cut-off for abdominal obesity^17^ the cut point for MetS indicator variables were considered as follows (1) WC ≥ 89 cm in males and ≥ 86 cm in females; (2) TG ≥ 150 mg/dL; (3) low HDL-cholesterol (< 40 mg/dL in males and < 50 mg/dL in females); (4) high blood pressure (BP ≥ 130/85 mm Hg) or on antihypertensive medication; and (5) FG ≥ 100 mg/dL or on antidiabetic medication.

### Physical activity (PA)

The intensity of physical activity was determined using the International Physical Activity Questionnaire (IPAQ). This questionnaire, consisting of 20 items, can measure routine physical activities of rural Iranians. The amount of each activity in hours and minutes was determined; the total physical activity score was calculated based on formula of MET coefficient of activity duration^14^.

### Sleep duration and quality

Sleep duration was measured as a continuous variable through the subtraction of two questions (“when do you usually go to bed?” and “when do you usually get up in the morning?”). The sleep duration was calculated in hours by subtracting wake-up time from bedtime and then a mean of night sleep duration per 24 h was calculated according to the participants’ answers. Data on sleep quality was collected through a binary variable asking the participants about the presence of any of the sleep disorders listed (use of sleep medication, limb movements during sleep and the difference between sleeping and waking time with the desired time)^14^.

### Social status

In this study, the social status of each person was assessed by considering their level of education and employment status. Social status is defined as a latent variable resulting from education and employment status in our model.

### Statistical analysis

A descriptive analysis of the main characteristics of interest was reported as frequencies, means, and standard deviations. Two independent sample test was used to *t* test for differences in general characteristics across different gender, while, Chi-square test was used to evaluate the distribution of qualitative variables between males and females.

**Figure 1:**
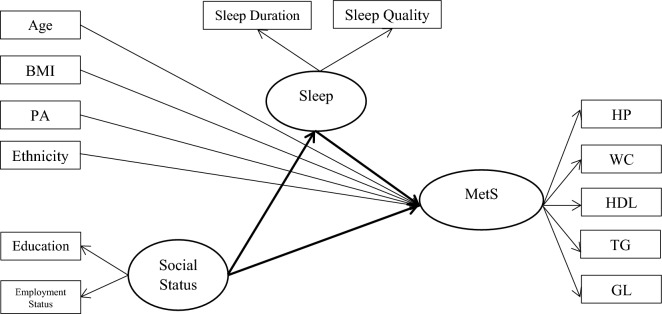
Hypothesized structural equation model for significant relationships between MetS and associated factors. Oval circles indicate latent variables which are not measured directly. MetS was implemented as a latent variable with five domains as predictors including *BMI* body mass index, *PA* Physical activity, *HP* Hypertension, *WC* Waist circumference, *HDL* high density lipoprotein cholesterol, *TG* triglycerides, *GL* glucose. Social status was implemented as a latent variable with two domains as predictors including Education and Employment Status. Sleep was implemented as a latent variable with two domains as predictors including sleep duration and sleep quality.

The primary outcome for the current study was evaluating the risk factors affecting MetS using SEM. SEM allowed us to assess multiple relationships across variables and measure MetS which cannot be directly observed. According to previous studies and based on the univariate analyses in the prior steps, 13 observed variables were selected to construct the hypothesized theory-driven model. In our model, the latent continuous variable, MetS, was loaded by incorporating the HP, WC, HDL, TG, and GL. Additionally, the sleep latent variable was represented by sleep duration and sleep quality indicators. Lastly, the social status latent variable was measured by education and employment status indicators. Confirmatory factor analysis (CFA) of MetS, social status, and sleep components was carried out. SEM was used to test the direct and indirect effects social status on MetS, mediated by sleep, controlled by confounding factors including age, BMI, ethnicity and physical activity (MET) (Fig. 1).

Since the MetS is different between males and females, the model was compared in both groups using multiple-group analysis to determine whether the estimates vary by gender. The method of estimation was Maximum Likelihood (ML) based on the covariance matrix. The model’s goodness of fit was examined using seven fit indices including relative chi-square (× 2/df), range 2 to 5, the comparative fit index (CFI) > 0.90, the goodness-of-fit index (GFI) > 0.90, and the root mean square error of approximation (RMSEA) < 0.07. Values for the adjusted goodness-of-fit (AGFI) of ≥ 0.90 and ≥ 0.95 indicate acceptable and good fit, respectively. Values for the Normed-fit index (NIF) of ≥ 0.95 represent good fit, whereas Non-Normed Fit Index (NNFI) values > 0.80 represent good fit^18^.

## Results

### Characteristics of subjects

Data was cleaned up and validated. Participation rate was almost 94% and declining to participate was due to unwillingness, lack of confidence and feeling unwell. The response rate was 99%. The analysis included data from 10,138 individuals (5558 (54.8%) females and 4580 (45.2%) males) with mean (SD) age of 48.2 (9.6) years. The average sleep hours were 7.9 ± 4.0 per day from which 6.5 ± 1.7 h (85% of participants) were night sleep. In addition, poor sleep quality was seen in 55.8% of participants.

Demographic, social status and health characteristics of the subjects, for both female and male, were significant differences in all MetS components between males and females (Table 1). Females had a significantly higher mean sleep duration and lower physical activity than males. There were 1337 (29.2%) males with three or more of the five possible MetS components and 2100 (37.8%) females.

**Table 1:** Comparison of characteristics of study participants by gender at baseline.

Variable	Category	Females (n = 5558)	Males (4580)	p-value
Age	–	48.3 ± 9.6	48.2 ± 9.7	0.788
Waist circumference (cm)	–	96.2 ± 11.5	89.5 ± 11.2	< 0.001
Triglyceride (Mg/dl)	–	128.8 ± 74.6	136.4 ± 91.2	< 0.001
Fasting blood sugar (Mg/dl)	–	94.9 ± 33.1	90.6 ± 24.3	< 0.001
Systolic blood pressure	–	112.7 ± 19.0	111.3 ± 17.4	< 0.001
Diastolic blood pressure (mmHg)	–	75.3 ± 11.9	74.8 ± 11.5	0.044
HDL-C (Mg/dl)	–	54.4 ± 16.5	47.7 ± 14.6	< 0.001
BMI	–	26.9 ± 4.8	24.2 ± 4.4	< 0.001
Sleep duration	–	8.1 ± 4.0	7.8 ± 3.8	< 0.001
Mother ethnicity	Fars	3006 (56.4)	2325 (43.6)	< 0.001
	Arab	324 (57.1)	243 (42.9)	
	Turk	2192 (52.7)	1971 (47.3)	
Father ethnicity	Fars	2817 (56.4)	2177 (43.6)	0.001
	Arab	365 (55.1)	298 (44.9)	
	Turk	2337 (53.1)	2062 (46.9)	
Education	Illiterate	1733 (68.4)	799 (31.6)	< 0.001
	Under diploma	3513 (54.2)	2964 (45.8)	
	Diploma	221 (24.9)	668 (75.1)	
	BS	50 (33.6)	99 (66.4)	
	MS or PhD	4 (19.0)	17 (81.0)	
Physical activity (MET)	Low	6 (40.0)	9 (60.0)	< 0.001
	Moderate	5176 (62.5)	3108 (37.5)	
	High	376 (20.4)	1463 (79.6)	
Quality of sleep	High	2245 (40.4)	2232 (48.7)	< 0.001
	Poor	3313 (59.6)	2348 (51.3)	
Employment status	Unemployed	4405 (87.6)	633 (22.6)	< 0.001
	Employed	1153 (22.6)	3947 (77.4)	
Smoking	No	5427 (66.3)	2761 (33.7)	< 0.001
	Yes	131 (6.7)	1819 (93.3)	
Use hookah	No	5454 (59.1)	3779 (40.9)	< 0.001
	Yes	104 (11.5)	801 (88.5)	
Use alcohol	No	5540 (60.4)	3636 (39.6)	< 0.001
	Yes	18 (1.9)	944 (98.1)	
B-blocker	No	4894 (53.1)	4328 (46.9)	< 0.001
	Yes	664 (72.5)	252 (27.5)	
Thiazide diuretics	No	5441 (54.6)	4533 (45.4)	< 0.001
	Yes	117 (71.3)	47 (28.7)	
Thiazolidinedione agent	No	5546 (54.8)	4574 (45.2)	0.312
	Yes	12 (66.7)	6 (33.3)	
Glucocorticoids	No	5431 (54.4)	4546 (45.6)	< 0.001
	Yes	125 (80.1)	31 (19.9)	
Lipid-lowering agents	No	5061 (96.2)	202 (3.8)	< 0.001
	Yes	497 (10.1)	4439 (89.9)	
Diabetes medications	No	5181 (53.9)	4439 (46.1)	< 0.001
	Yes	377 (72.8)	141 (27.2)	

Independent sample *t* test and Chi-square test were used to compare quantitative and qualitative variables between males and females respectively. *HDL-C* high-density lipoproteins cholesterol, *BMI* body mass index, *MET* metabolic equivalent.

### Structural equation modelling

The correlation coefficients among the manifest variables for examining the association between associated variables and MetS were estimated in females and males. All of the MetS components were statistically correlated with social status variable, sleep, age, BMI, ethnicity and physical activity.

The CFA of Fig. 1 resulted in a good model fit. The selected indicators loaded significantly on MetS, social status, and sleep components. Most changes in MetS are described by the WC index in both female and male; after WC, HP and TG were the two most important MetS components in females and TG and GL in males. In the male group 57.8% and in the female group 45.3% changes in the MetS are explained by the WC index. Most changes in social status, and sleep components are described by employment status and quality of sleep respectively in both gender (Table 2).

**Table 2:** The confirmatory factor analysis results of the structural equation model.

Latent variable	Factors	Factor loadings			
		Males		Females	
		Standardized Effect	Proportion of variance accounted for	Standardized Effect	Proportion of variance accounted for
Metabolic syndrome (MetS)	HP	0.306	0.094	0.409	0.167
	WC	0.760	0.578	0.673	0.453
	HDL	− 0.149	0.022	− 0.064	0.004
	TG	0.339	0.115	0.333	0.111
	GL	0.331	0.109	0.209	0.043
Social	Education	0.345	0.119	0.102	0.010
	Employment status	0.581	0.338	0.135	0.018
Sleep	Duration	0.375	0.141	0.432	0.187
	Quality	0.422	0.178	0.524	0.274

*HP* hypertension, *WC* waist circumference, *HDL* high-density lipoproteins, *TG* triglyceride, *GL* fasting.

Multiple-group SEM revealed significant differences between males and females in path coefficients (p < 0.05).

In both male and female, the model fits the data well $(\chi 2/df$ = *3.6, CFI* = *0.92, GFI* = *0.92,* RMSEA = 0.060 [0.054–0.67]) (Table 3). Based on the results, social status, controlled by factors such as age, BMI, physical activity, and ethnicity, has a direct negative impact on the occurrence of MetS in both male and female. Additionally, sleep acts as a mediator, with a significant negative effect on MetS which leads to a significant positive indirect effect of social status on the development of MetS (in both male [direct effect = − 0.212, P = 0.014; indirect effect = 0.004, P = 0.002 and total effect = − 0.208, P = 0.011] and female group [direct effect = − 0.625, P = 0.028; indirect effect = 0.004, P = 0.002 and total effect = 0.621, P < 0.001]).

**Table 3:** Maximum-likelihood parameter estimates for the structural regression model, metabolic syndrome (MetS).

	Males			Females		
	Estimate	S.E	P-value	Estimate	S.E	P-value
Direct effect						
Age → MetS	0.129	0.001	< 0.001	0.535	0.003	< 0.001
BMI → MetS	0.915	0.002	< 0.001	0.631	0.009	< 0.001
Ethnicity → MetS	0.011	0.001	< 0.001	0.014	0.001	< 0.001
Physical activity → MetS	− 0.067	0.005	< 0.001	− 0.022	0.004	< 0.001
Social → MetS	− 0.212	0.014	< 0.001	− 0.625	0.028	< 0.001
Sleep → MetS	− 0.072	0.024	0.003	− 0.095	0.039	0.005
Social → Sleep	− 0.055	0.001	< 0.001	− 0.041	0.001	< 0.001
Indirect effect*						
Social → Sleep → MetS	0.004	0.002	0.009	0.004	0.002	0.009
Total effect*						
Social → MetS	− 0.208	0.011	< 0.001	− 0.621	0.011	< 0.001
$\frac{{{\varvec{\chi}}}^{2}}{{\varvec{d}}{\varvec{f}}}$	3.6					
RMSEA	0.060					
GFI	0.92					
AGFI	0.84					
CFI	0.92					
NFI	0.90					
NNFI	0.91					
IFI	0.92					

Direct effects are depicted in Fig. 1.

*MetS* metabolic syndrome, *BMI* body mass index, *RMSEA* root mean square error of approximation, *GFI* goodness-of-fit index, *AGFI* adjusted goodness-of-fit index, *CFI* comparative fit index, *NFI* normal fit index, *NNFI* negative normal fit index, *IFI* incremental fit index.

*Bootstrap estimates under bias-corrected percentile method.

Based on the results, in both male and female, age, ethnicity, and BMI have a significant positive effect on the occurrence of MetS while physical activity shows a significant negative effect. In both groups, BMI has the greatest effect on the occurrence of the MetS (the path coefficient is 0.915 for males and 0.631 for females). The effect of BMI on the incidence of MetS in males is more than females. In males, age, social status, sleep, and physical activity, respectively, have the greatest role in explaining the variance of MetS after BMI. However, in females, social activity, age, sleep, and physical activity, respectively, have the greatest role in explaining the variance of MetS (Table 3).

## Discussion

The main purpose of this study was to use a structural equation model (SEM) to firstly estimate and identify the impact of different MetS components separately for females and males considering MetS as a latent continuous variable rather than a binary variable, and secondly to evaluate the interrelations among the associated factors involved in the development of MetS separately for females and males. Therefore, we created the theory-based path-diagram and used the SEM to test the hypothesized relationships proposed in the theory-based path-diagram.

The prevalence of metabolic syndrome has also been evaluated in this study. This prevalence was 28.3% (32.1% of females and 24.7% of males) that indicated a higher prevalence of MetS in females. The prevalence of MetS has been different in different studies, which might be due to the application of different definitions for this syndrome. In a meta-analysis of 125 Iranian studies (105 cross-sectional and 20 cohort studies) the pooled prevalence of MetS was 27% based on the International Diabetes Federation (IDF) criteria and the ATP III criteria and MetS were more frequent in females (34%) than in males (22%)^19^; several other studies and meta-analysis^11,20–26^ have also reported a higher prevalence of MetS in females. Some studies have also shown that the prevalence of MetS is higher in females after their fifth decade of life and also after menopause^22,24,27,28^. For example, a large-scale study of 36 cohorts (the MOnica, Risk, Genetics, Archiving and Monograph—MORGAM project) reported the prevalence of MetS increased among adults from age group 19–39 years to 60–78 years two-fold in males and five-fold in females^29^. Considering the average age of the female population in this study) 48.3 ± 9.6(, the following factors may have an impact on the higher prevalence of metabolic syndrome in female participants of this study. According to the articles hormonal differences between males and females play an important role in the development of metabolic syndrome^30,31^. According to studies, female estrogen levels decline during menopause, although estrogen has many beneficial effects on the body, including improving insulin sensitivity, reducing inflammation, and promoting the breakdown of fats^30–32^, but a decline in estrogen levels can lead to an increase visceral abdominal fat, which is associated with insulin resistance, elevated free fatty acids, increase in WC which can significantly affect blood preacher and increased hepatic lipase activity^33^ and also due to physiological changes during this time studies have also shown that while females tend to gain subcutaneous adipose tissue with age regardless of menopausal status, the increase in visceral adipose tissue only occurs after menopause and is associated with a decline in 17β-estradiol^34,35^ which may increase their risk of developing metabolic syndrome. In addition, considering the rurality of our study area, they may face socio-economic challenges such as lower income levels, higher poverty rates, and limited educational opportunities especially for females that contributing to a higher prevalence of metabolic syndrome.

In contrast, a recently published study of the American population (aged ≥ 20 years), using a US nationally representative sample of 17,048 individuals, showed that the overall prevalence of MetS was 35.1% in males and 34.3% in females; this result showed that MetS prevalence was not significantly different among American males and females^36^.

Following the main purpose of this study, results from the SEM showed that in females most changes in MetS are described by WC, followed by HP and TG, while in males most changes in MetS are described by WC, followed by TG then GL. The results of this study indicate that 45.3% and 57.8% changes in the MetS are explained by the WC index in females and males respectively. Accordingly, WC has the highest factor loading indicating that WC is the most important component of MetS in both female and male. This could be explained by the fact that WC is closely associated with insulin resistance due to its correlation with visceral fat quantity. In centrally obese adults proinflammatory adipocytokines and excess fatty acids are released into the portal circulation by visceral adipocytes and leads to increasing hepatic steatosis and insulin resistance^37^. In general, therefore, in a multi-ethnic country like Iran it is necessary to have a country-specific or even ethnic-specific WC cut-off points.

According to the MetS variance explained, another important component of MetS in both female and male was TG. There is evidence indicating the important role of TG in insulin resistance^38–40^; for that reason, a considerable amount of literature has investigated on hypertriglyceridemia waist phenotype as a simple and cost-effective measurement for screening MetS and MetS-related diseases^40–42^.

In this study in both female and male, WC index has the highest impact among the MetS components while in another study focusing on Iranian urban residents, hypertension explained the most changes of MetS^43^. This different result could be due to the rural lifestyle and environmental conditions in the current study. Urbanization is associated with decreased physical activity^44^ and higher intake of processed food^45^, increased risk of chronic disease (type 2 diabetes, hypertension and the MetS)^44,46–48^ and psychosocial stress^48,49^. As rural areas are less affected by artificial environmental conditions, the MetS components’ factor loadings are more likely to reflect the natural physiological relationships in our recruiting participants.

Results from the SEM confirmed the gender effects of social status on MetS, mediated by sleep and controlled by age, BMI, ethnicity and physical activity. This demonstrated that after controlling for age, BMI, ethnicity and physical activity, individuals (both male and female) with a higher level of social status (those employed with higher education level) were at lower risk for MetS. This association was mediated by sleep and could be explained by the fact that stricter schedule and regularity for sleeping hours in participants who are employed could lead to lower risk of for MetS because sleep–wake cycle and circadian system, which is linked to both sleep and metabolism, is less frequently disturbed in employed participants than unemployed ones^50,51^.

Current study also demonstrated an inverse correlation between sleep (sleeping hours and sleep quality) with the prevalence of MetS. Several epidemiological studies have investigated the association between sleeping hours and MetS, with contradictory results. Some of them have reported that short sleep duration was significantly associated with the risk of MetS^52–56^, while conversely several studies showed that long sleep duration was associated with MetS^57,58^. Furthermore, some studies have reported a U-shaped association between sleep duration and MetS as both short and long sleeping hours were associated with the risk of MetS^58,59^. A study conducted on the Fasa PERSIAN cohort study reported a correlation between short sleep duration and higher prevalence of MetS components such as CVD, and hypertension and indicated an inverse correlation between sleep duration, age, and BMI^60^. Several pathophysiologic mechanisms link short sleep duration and MetS. Experimental studies have shown that^61–63^, sleep restriction can reduce leptin and increase ghrelin levels, leading to increased hunger and appetite specifically for carbohydrate-dense food^63^. The deficiency in melatonin secretion, as in shift-work associated with reductions in blood melatonin levels^64^ .Recent studies indicates that melatonin has potential benefits for cardiovascular health and metabolic syndrome by influencing on blood pressure regulation, lipid metabolism, and glucose homeostasis^64,65^. In addition, chronic sleep insufficiency can cause an increase in both in advanced glycation end products (AGEs)^66^ and pro-inflammatory cytokines, both of which can contribute to an increase in insulin resistance^67^. In general, human studies has shown the chronic sleep insufficiency can cause an imbalance of adrenalin, cortisol and glucose metabolism and cause increase in appetite, adiposity, weight gain, inflammation and endothelial damage^68,69^.

One of the notable findings from the present study was that an increased level of BMI leads to an increase in MetS occurrence. Previous studies also have demonstrated similar findings with this study. According to these studies, MetS occurs most commonly in individuals with a BMI higher than 30 kg/m^2^^70,71^. Obesity has been shown to be a strong predictor of the occurrence of MetS by predisposing to diabetes, hypertension, and hyperlipidemia^72–75^. Also, our results demonstrated that men with higher level of BMI are more susceptible to MetS compared to females. This could be due to sex differences in central obesity distribution. females are more prone to peripheral adiposity; central obesity is more common in males^33,76^. Centrally accumulation of body fat is associated with increased the development of type 2 diabetes and cardiovascular mortality^76–78^. Visceral fat produces free fatty acids and a variety of factors known as adipokines. These adipokines include hormones (e.g. leptin and adiponectin) and inflammatory cytokines (e.g. tumor necrosis factor α (TNFα), interleukin (IL)-6, omentin and visfatin) and other proteins (e.g. plasminogen activator inhibitor (PAI)-1, angiotensinogen, resistin and apelin)^79^. The inflammatory mediators and FFA is directly delivered to the liver via the portal vein and may contribute to the health problems associated with adiposity such as dyslipidemia, insulin resistance and atherosclerosis^79–83^. Considering WC as a good surrogate marker of visceral fat accumulation beside higher visceral fat accumulation in males^33^, could explain our results indicating that 57.8% of MetS changes in males are explained by the WC index while this impact rate is 45.3% in the female’s group.

Current study indicated that individuals with a higher social status (higher education and being employed) were less susceptible to the incidence of MetS. Previous studies have also reported an inverse association between social condition and developing MetS^84–89^. According to those studies, the prevalence of MetS is higher among the unemployed and even housewives, and they also reported an inverse relationship between education level and MetS. These results showed that higher level of education can lead to greater awareness of a healthy lifestyle. Individuals with higher education have also a better economic situation. Higher level of income can lead to an increase in quality of life and prevent the prevalence of MetS risk factors. This contrary relationship between social status and the prevalence of MetS among the Iranian population has also been reported in previous studies^90,91^. Another finding from this study was that females with lower social status are more susceptible to increase prevalence of MetS compared with males, which was consistent with previous studies^92,93^. This could be explained by the fact that in rural areas males with lower social status are more likely to have manual occupations which reduces the risk of MetS.

One of the strongest predictors of MetS is age^29,94,95^. The large increase in the prevalence of MetS in older adults can be explained by a lifetime accumulation of risk factors^95–97^ consistent whit these studies our findings show that MetS risk factors typically develop or worsen with advancing age in both genders, but it influences females more strongly. The present findings seem to be consistent with K. Vishram study using 36 cohorts from the MORGAN-Project with 69,094 participants which reported that aging can increase MetS prevalence by 5 and 2 times in females and males, respectively^98^. A meta-analysis that was conducted on the Iranian population reported that in the age group of older than 40 years (compared to those under 30) the frequency of the MetS was significantly higher in the female group^11^. This, gender differences in older females can be due to sex hormone changes and increased blood pressure (BP) in postmenopausal females, which can eventually lead to cardiovascular disease^98,99^.

Our results indicated that hypertension is the second most important MetS component in females whereas the fourth in males. This could be explained by the fact that with ageing the rate of hypertension increases faster in females than males^100^. The incidence of hypertension is reported to be higher in postmenopausal females than in premenopausal females^101,102^. After menopause, the WC also increases, which can significantly affect BP level^102^. In centrally obese hypertensive females and after menopause accumulated visceral fat is reported to be associated with higher blood pressure levels and insulin resistance^103^. Several mechanisms have been identified to explain how obesity is associated with hypertension. Renin–angiotensin–aldosterone system (RAAS), the sympathetic nervous system (SNS), endothelial dysfunction, hyperinsulinemia, adipokine imbalance, and increased inflammatory cytokines, increase in intraabdominal pressure, glomerular and tubular effects^103–105^. Some of these mechanisms (e.g. renin angiotensin system) are differently regulated in males and females^106^. Another possible explanation for stronger impact of hypertension component of MetS in females than males might be that postmenopause females are more salt sensitive in their blood pressure regulation than premenopausal females^107^.

In addition, our result indicated the effect of ethnicity on the MetS risk factor. Previous studies have examined the prevalence of MetS in different ethnicities and found that this prevalence varies among different ethnicities^108,109^. Despite differences in diagnostic criteria, age, and gender of subjects, many studies have investigated the prevalence of MetS in different ethnic groups in Iran as well^110–124^. According to these articles, the prevalence of MetS varies among different Iranian ethnic groups. Among these, the highest prevalence of MetS has been reported in studies such as Khorasan Razavi and Bushehr studies^125,126^. In contrast, the lowest rat e has been reported in Isfahan and Khorasan studies^26,123^. In general, in a meta-analysis of 69 studies, Kalan Farmanfarma et al. reported that the prevalence of MetS was higher in southern (Boshehr and Hormozgan) and northern (Mazandaran) Iran than elsewhere^11^. Based on the current study, which was conducted on the population of the Fasa PERSIAN cohort study (including three ethnicities, Persian, Turkish, and Arab), it was found that ethnicity plays an important role in the risk of developing this syndrome. So that risk of metabolic syndrome was higher in Arab and Turkish people compared to Persian ones. Based on local research in this area it was found that the lifestyles and eating habits of the three ethnic groups of Persia and Turkish and Arab are clearly different. Arab and Turkish ethnicities in this region have sweet and high-fat diet as well as high-calorie lifestyles, which can lead to increased risk factors for metabolic syndrome.

Another variable that had a significant relationship with MetS in this study was MET score as a measure for total physical activity. According to our results 62% of the males and 79% of the females had moderate physical activity, and 20% and 37.5% of them had high physical activity respectively. The studies indicated that physical activity is helpful to reduce the MetS risk factors^86,127–129^. But some of them have shown that only a high level of physical activity is associated with a lower risk of MetS^130,131^. Physical activity can reduce the MetS risk factors such as high blood pressure, low HDL-C, elevated fasting glucose, and abdominal adipose tissue accumulation^127,132–137^. Consistent with these studies, the present study also showed an inverse correlation between MET that the prevalence of MetS, so that with increased physical activity, the prevalence of this syndrome decreases.

This study has some limitations; firstly, the collected data in the field of sleep and physical activity, and social status were based on self-reported by the individuals. Secondly, this study was a cross-sectional study and we used one-time measurement points that adding new follow-up can increase the value of the study. Thirdly Sleep quality measured in this study was not based on global sleep quality protocols and was measured according to three questions (mentioned before) which showed each of these three factors increased the risk of MetS, therefore, the results are not comparable to other studies.

## Conclusion

In conclusion, the results of this study, which were performed on a large population of Fasa PERSIAN cohort study using SEM, indicate that WC has the highest factor loading and is identified as the most important component of MetS explaining 45.3% and 57.8% of changes in the MetS in females and males respectively. In general, therefore, in a multi-ethnic country like Iran it is necessary to have a country-specific or even ethnic-specific WC cut-off points. Results from the SEM also showed that in females most changes in MetS are described by WC component, followed by HP and TG, while in males most changes in MetS are described by WC, followed by TG then GL. Therefore, the integration of TG and WC within genders could be useful as a screening criteria for MetS in our study population. Results from the SEM also confirmed the gender effects of social status on MetS, is mediated by sleep and controlled by age, BMI, ethnicity and physical activity. Age, sex, ethnicity, sleep, SES, and physical activity are also found to be associated with the prevalence of MetS.

## Data Availability

The datasets used and/or analyzed during the current study available from the corresponding author on request.
